# Resting state brain connectivity of pain and fatigue is affected by performance of a fatiguing attention task in patients with chronic pain

**DOI:** 10.1016/j.ynirp.2026.100372

**Published:** 2026-06-10

**Authors:** Nils Berginström, Marika C. Möller, Anna Holmqvist, Monika Löfgren, Britt-Marie Stålnacke, Love Engström Nordin

**Affiliations:** a Department of Community Medicine and Rehabilitation, Rehabilitation Medicine, Umeå University, Umeå, Sweden; b Department of Psychology, Umeå University, Umeå, Sweden; c Department of Clinical Sciences, Karolinska Institutet, Stockholm, Sweden; d Department of Rehabilitation Medicine, Danderyd University Hospital, Stockholm, Sweden; e Department of Neurobiology, Care Sciences and Society (NVS), Karolinska Institutet and Department of Diagnostic Medical Physics, Karolinska University hospital, Solna, Stockholm, SE-171 76, Sweden

**Keywords:** Chronic pain, Fatigue, Resting state functional connectivity, Functional magnetic resonance imaging

## Abstract

Fatigue is common in patients with chronic pain, but the neural underpinning of this symptom is still poorly understood. In this study, we aimed to investigate resting state connectivity correlates of pain and fatigue in patients with chronic pain. Twenty-four women with chronic pain, and 22 age-matched healthy controls underwent resting state functional magnetic resonance imaging (fMRI), performed a 20-min fatiguing attention task, and underwent an additional resting state fMRI examination. Patients with chronic pain reported a higher degree of both fatigue (p < 0.001) and pain (p < 0.001) than healthy controls. Significant group differences in connectivity to the lateral visual network, the salience network, the auditory network, the executive control network, and especially in regions within the default mode network were detected before the task. After the task, these differences were no longer statistically detectable, and only differences in connectivity between the basal ganglia network and the prefrontal areas between groups were observed. While differences in functional connectivity related to pain were present before performance of the task, only differences related to fatigue were found after the task. This finding indicates that processing of fatigue might overshadow processing of pain after performance of a fatiguing attention task in patients with chronic pain.

## 1 Introduction

Chronic pain is defined as pain lasting longer than 3 months (International Association for the Study of Pain, 2020), and is a common public health problem (Breivik et al., 2006). Besides the pain in itself, comorbidities and other symptoms such as depression (Bair et al., 2003), sleep difficulties (Cohen et al., 2021), and cognitive dysfunction (Moriarty and Finn, 2014; Moriarty et al., 2011) are frequently reported in patients with chronic pain. One of the most common and disabling symptoms in patients with chronic pain is fatigue, with a prevalence exceeding 50% of patients with chronic pain (Creavin et al., 2010; Goertz et al., 2021). Fatigue is a multidimensional concept that can be defined as both a transient condition (state fatigue), and stable state fatigue in everyday life (trait fatigue) (Genova et al., 2013). While prevalent in several chronic pain conditions (Boggero et al., 2022; Dailey et al., 2016; Murphy et al., 2016; Nikolaus et al., 2013; Park et al., 2024) the link between pain and fatigue in patients suffering from chronic pain is still unclear (Van Damme et al., 2018; van Dartel et al., 2013). Analyzing the correlations between the brain and manifestations of symptoms can facilitate in explaining this relationship.

Resting state functional connectivity is a technique utilizing the fact that the brain at rest shows low frequency fluctuations (<0.1 hz) of the blood-oxygen level-dependent (BOLD) signal (Raichle et al., 2001). By investigating correlations between regions in this signal, functional brain networks can be detected. Chronic pain has been extensively investigated in relation to these networks. Alterations within the default mode network (DMN) are consistently observed in patients with chronic pain (Basu et al., 2018; Hemington et al., 2016; Ichesco et al., 2014; Liu et al., 2020; Napadow et al., 2010; Schrepf et al., 2018). Both increased and decreased intrinsic and extrinsic connectivity have been observed, depending on the diagnostic group examined and pain chronicity (see Pfannmoller and Lotze (2019) for a review). The insula, a region within the salience network (SN), plays an important role in evaluating internal states. As such, the insula has been found to have altered connectivity in patients with chronic pain, both increased connectivity to the DMN (Basu et al., 2018), and decreases in connectivity to the sensory motor network (Flodin et al., 2014) and to the frontal cortices (Ichesco et al., 2014; Kim et al., 2017). Findings on connectivity within the SN, e g between the insula and the cingulate cortex, are mixed in patients with chronic pain (Cifre et al., 2012; Ichesco et al., 2014, 2016; Kim et al., 2017).

Fatigue in patients with chronic pain has not been very well studied using resting state functional magnetic resonance imaging (fMRI). Liu et al. (2020) examined patients with ankylosing spondylitis and low back pain, using resting state fMRI and reported alterations in several networks as described above. Both self-reported pain intensity and trait fatigue were associated with increased functional connectivity between insula and medial prefrontal cortex as well as increased connectivity between the left and medial prefrontal cortex, whereas only self-rated pain was related to decreased functional connectivity within the DMN. Basu et al. (2018) found that increased DMN-insula connectivity was associated with a higher degree of self-assessed trait fatigue, but they did not find a significant relationship between state fatigue and functional connectivity in patients with rheumatoid arthritis. Thus, state and trait fatigue may have distinct neural correlates in patients with chronic pain. While some studies have revealed relationships between self-assessed trait and/or state fatigue and functional connectivity in patients with chronic pain (Ichesco et al., 2014; Liu et al., 2020), others have not (Hemington et al., 2016; Park et al., 2022). Thus, the neural correlates of fatigue in patients with chronic pain remain unclear (Goni et al., 2018).

Empirical evidence on fatigue in patients with neurological conditions suggests that fatigue may arise from dysfunction within striato-thalamic cortical loops within the basal ganglia network (BGN) (Chaudhuri and Behan, 2000, 2004). Studies using fatiguing tasks to alter resting state networks in patients with traumatic brain injury have shown that networks involving the striatum and thalamus are affected by state fatigue eliciting tasks (Bruijel et al., 2022; Nordin et al., 2016). Considering these associations to fatigue, and the established role of the DMN, the SN, and the BGN in pain processing (Apkarian et al., 2005; Borsook et al., 2016; Tracey and Mantyh, 2007), particular attention should be directed toward these networks when examining fatigue in individuals with chronic pain. To our knowledge, no study has previously examined manipulations of resting state networks through fatiguing tasks in patients with chronic pain.

The primary aim of the present study was to investigate changes in resting state functional brain connectivity from before to after a fatigue eliciting task in patients with chronic pain in comparison to healthy controls (group × time interaction). We hypothesized that including a fatiguing vigilance task would induce changes within the BGN in patients but not in healthy controls. Another aim was to investigate the correlations between resting state functional connectivity and trait and state fatigue and pain within these groups. We hypothesized that there would be significant resting state functional connectivity differences between patients with chronic pain and healthy controls within the DMN and the SN, and that self-assessed pain and fatigue (state and trait) would be associated with functional connectivity within the DMN/SN and the BGN, respectively.

## 2 Materials and methods

### 2.1 Participants

All patients referred to the pain rehabilitation clinic at Umeå, University Hospital, Sweden, from September 2020 to September 2021 were asked to participate in this study. Inclusion was based on chronic pain, which was defined as pain debut >3 months earlier. Only right-handed female participants were included, and inclusion age ranged from 18 to 45. The exclusion criteria were as follows: Acquired brain injury (including concussion); any neurological condition; severe psychiatric disorder (such as psychotic disorders, bipolar disorder, or severe depression); intellectual disability; pregnancy; non-speaker of Swedish; and medications that could impact cognitive functioning (e.g., sedatives, opioids). Data regarding menstrual cycle of participants was not recorded.

Sample size was based on a previous study from our research group including patients with mild traumatic brain injury (Nordin et al., 2016). Among the 95 patients who were screened for eligibility, 48 patients declined to participate, and 2 were unreachable. A total of 45 patients agreed to participate, but 21 were excluded due to fulfilling one or several exclusion criteria, mainly concussion, not speaking Swedish or medical treatments interfering with cognitive function.

Healthy controls were recruited through flyer posting at the University Hospital of Umeå. A total of 39 healthy controls expressed interest in participating in the study, and 22 were included to match the patient sample as closely as possible with respect to age and level of education. The inclusion and exclusion criteria were the same for healthy controls as for the patients, except that controls were also excluded if they had any current pain or a history of chronic pain (>3 months).

### 2.2 Instruments

#### 2.2.1 Demographic data and self-assessment scales

Demographic data and results from self-assessment scales for patients were obtained from the Swedish Quality Registry for Pain Rehabilitation (SQRP: http://www.ucr.uu.se/nrs). The SQRP is a national registry to which most specialist pain care centers in Sweden deliver data on various aspects of pain and other health-related variables from their patients. In the present study, several variables regarding pain (years since pain debut, Numeric Rating Scale (pain during the last week), and number of pain localizations) from the SQRP were included, as were the self-assessment questionnaires the Hospital Anxiety and Depression Scale (HADS) (Zigmond and Snaith, 1983) and the Insomnia Severity Index (ISI) (Bastien et al., 2001). All participants also completed the Multidimensional Fatigue Inventory (MFI-20) (Smets et al., 1995), a measure of trait fatigue. In addition, all participants completed visual analogous scales (VAS) on state fatigue (VAS-f) and pain (VAS-p) before and after the MRI examination, which ranged from 0 (no fatigue/pain at all) to 100 (worst possible pain/fatigue).

### 2.3 Procedure

The study protocol was approved by the Swedish Ethical Review Authority (no 2018/424-31) and was performed in adherence to the Declaration of Helsinki. The study protocol has been previously published (Moller et al., 2023) and registered at clinicaltrials.gov (trial registration no: NCT05452915). Patients completed the SQRP questionnaires within one month before entering this study. All data collection was performed before the patients had undergone any specialized pain rehabilitation. Healthy controls completed all self-assessment scales and responded to questions regarding demographic data in adjacent to undergoing the other examinations.

All participants arrived having fasted beforehand for blood sample collection (reported elsewhere) at 7:45 in the morning. Before any examinations, they received written and verbal information about the study and signed informed consent. After the blood sample (not reported here) had been collected, participants were offered breakfast, including coffee or tea, before undergoing MRI examination. The MRI session started at 9:00 and lasted approximately 1 h. After initial localizer volumes, scanning started with the first resting state fMRI session. This was followed by a task fMRI that included an attention task to induce state fatigue in participants, the Psychomotor Vigilance Task (PVT), which has been previously described (Möller et al., 2017; Nordin et al., 2016). In short, participants viewed a computer screen displaying a rectangle where four digits appeared. They were instructed to press a button with their right index finger as quickly as possible if the number 0 appeared but to withhold response for all other digits. The number was presented for 1 s, with a randomized inter-stimulus-interval of 2 to 10 s. Participants were given feedback after each correct answer of their reaction time, or “False answer” following an incorrect response or “No answer” if no answer was given within 1 s. The test lasted for 20 min. The results from the task fMRI and the results from the PVT are reported by Holmqvist et al. (2025), indicating that patients with chronic pain show increased reaction time during the duration of the task (i e fatigability), while healthy controls show stable reaction times during the duration of the task. After the task, the second resting state fMRI session was performed, followed by structural imaging acquisition (described in detail below).

#### 2.3.1 MRI procedure

All magnetic resonance images were acquired using a 3T General Electric Discovery MR 750 (General Electric, Milwaukee, Wisconsin) with a 32-channel-phased array head coil. A T2-weighted single-shot echo-planar imaging sequence sensitive to BOLD-contrast was acquired for resting state fMRI data before- and after the task fMRI. The sequence included 170 vol with 37 axial slices acquired in approximately 5:40 min. The following parameters were used: slice thickness of 3.4 mm and a slice gap of 0.5 mm in an interleaved order, echo time (TE)/repetition time (TR) = 30/2000 ms, flip angle (FA) = 80°, field of view (FOV) = 250 × 250 mm and matrix 128x128. During resting state, participants were instructed to look at a white fixation cross on a dark screen, while remaining awake. All participants were asked if they fell asleep during image acquisition.

### 2.4 Statistical analyses

#### 2.4.1 Behavioral data

Differences between groups were investigated using independent-samples t-tests for continuous variables, and chi^2^ tests for categorical variables. In analyses adjusted for differences in age and education, ANCOVAs were used. To investigate differences between groups in changes in self-assessment ratings of pain and state fatigue before and after fMRI, repeated-measures ANOVAs were used. If Mauchly's test for sphericity was significant, the Greenhouse-Geisser correction was performed. Post-hoc analyses were performed using paired samples *t*-test. Apha level was set to p < 0.05 for all statistical tests. All analyses were performed in SPSS Statistics 28.0 (IBM Corporation, Armonk, New York).

#### 2.4.2 fMRI

The resting state fMRI data was preprocessed using FMRIB's Software Library, FSL (http://www.fmrib.ox.ac.uk/fsl). The first five time frames in each data set were removed to ensure a steady state signal. Intra-modal motion correction was carried out using MCFLIRT (Jenkinson et al., 2002), which applies rigid-body transformations to correct head motion in the fMRI time series. No volume-wise censoring was performed. Instead, datasets showing motion exceeding the predefined thresholds were excluded entirely from further analyses to preserve sufficient resting-state scan duration. Translational motion parameters were inspected for all participants to ensure that head displacement did not exceed one voxel size (2 mm) to minimize residual motion-related effects not fully corrected by rigid-body realignment. For rotation parameters, a corresponding threshold of 0.04 radians was applied, derived by dividing the displacement limit by an assumed brain radius of 50 mm (θ = s/r = 2/50) (Power et al., 2012). Non-brain tissue was removed using brain extraction tool, BET (Smith, 2002). Spatial smoothing of 5 mm FWHM was applied to reduce noise. Spatial normalization to the standard MNI template was performed using a 12-parameter affine transformation and mutual-information cost function. During the affine transformation the imaging data was re-sampled to isotropic resolution using a Gaussian kernel with 4 mm full width at half maximum (FWHM). Independent Component Analysis (ICA) was performed using FSL's MELODIC (version 3.15) with the multisession temporal concatenation approach (Beckmann and Smith, 2005). ICA was run across all participants to achieve robust estimation of group-level components representing common spatial patterns of activity, thereby minimizing group bias. The number of output components was limited to 20 to avoid overfitting. A temporal high-pass filter with a cutoff of 100 s (0.01 Hz) was applied during preprocessing. No additional voxel-wise nuisance regression of motion parameters, white matter, cerebrospinal fluid (CSF), or global signal was performed prior to ICA decomposition. Instead, artifact-related components reflecting motion, white matter, CSF, or physiological noise were identified by visual inspection of spatial maps, time courses, and power spectra and excluded from further analyses. Individual estimates of the group level spatial maps were calculated using the tool Dual regression (Christian et al., 2009). First, for each subject, the group-average set of spatial maps was regressed into the subject's 4D space–time dataset. This results in a set of subject-specific time series, one per group-level spatial map. Next, those time series were regressed into the same 4D dataset, resulting in a set of subject-specific spatial maps, one per group-level spatial map. Contrast matrices were created in FSL's general linear model tool to perform two-way analysis of group and time. We tested for group- and time differences using FSL's randomize (Winkler et al., 2014) permutation-testing tool with 10,000 permutations in order to correct for multiple comparisons. After randomization, the results were thresholded at p < 0.05 (family-wise error corrected) using Threshold-Free Cluster Enhancement (TFCE). While a minimum cluster size of 2 contiguous resampled voxels (16 acquired voxels) was used for reporting, the use of TFCE ensures that significance is determined by both local signal intensity and spatial extent without the need for an arbitrary initial cluster forming threshold. We also tested for within group effects using self-assessment of depression (HADS) as well as trait fatigue (MFI) and state fatigue estimates (VAS-f) before and after MRI, and trait pain (Pain during the last week) and state (VAS-p) pain estimates before and after MRI as regressors. Differences from before to after MRI scanning were also analyzed regarding both VAS scales.

## 3 Results

### 3.1 Demographics and self-assessment scales

There were no significant differences in age between patients with chronic pain and healthy controls. However, healthy controls had a higher level of education (*p* = 0.003) and were more likely to be employed full-time (*p* < 0.001). There were no differences between groups regarding cognitive reserve/premorbid functioning as measured by Matrix Reasoning. Additionally, patients reported significantly higher levels of trait fatigue, insomnia, anxiety, and depression symptoms on all self-assessment scales (all *ps* < 0.001, see Table 1).

**Table 1:** Demographic data and self-assessment scales.

	Chronic Pain (n = 24)		Healthy controls (n = 22)		p
	M	SD	M	SD	
Age	33.38	7.94	29.77	6.41	0.10
Years of education	13.46	1.99	15.16	1.58	0.003
Multidimensional Fatigue Inventory	76.88	9.44	39.36	10.11	<0.001
Insomnia Severity Index	14.48	5.62	4.23	2.25	<0.001
HADS Anxiety	8.75	4.32	5.55	3.17	<0.001
HADS Depression	7.96	2.90	1.32	1.36	<0.001
Matrix Reasoning	19.36	3.62	18.46	4.33	0.448
Years since pain debut	8.32	5.97			
NRS Pain during the last week	6.48	1.38			
Number of pain localizations	18.33	8.64			

Note: HADS = Hospital Anxiety and Depression Scale. NRS=Numeric Rating Scale.

**Fig. 1:**
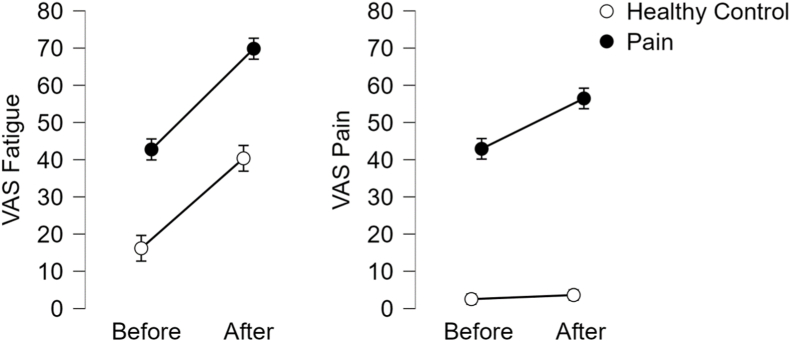
Self-assessment of fatigue and pain before and after fMRI.

Regarding self-reported fatigability, e.g. changes in state fatigue from before to after performance of the fMRI task, there were both a significant main effect of time (*p* < 0.001) and group (*p* < 0.001). The group analysis survived adjustment for differences in age and education. However, there was no significant group vs time interaction (*p = *0.645, Fig. 1).

For self-assessment of pain before and after fMRI there were also main effects of both group (*p < *0.001) and time (*p = *0.002). Additionally, there was a significant interaction effect of time vs group (*p* = 0.008), indicating that patients' pain increased from before to after fMRI, while the pain of healthy controls remained stable (Fig. 1) Both group effect and the interaction effect survived adjustment for differences in age and education. While the change in pain was significantly correlated to the change in state fatigue in the healthy controls (*r* = 0.43, *p* = 0.045), the same was not true for patients (*p* = 0.477).

### 3.2 Resting state fMRI data – functional connectivity

**Fig. 2:**
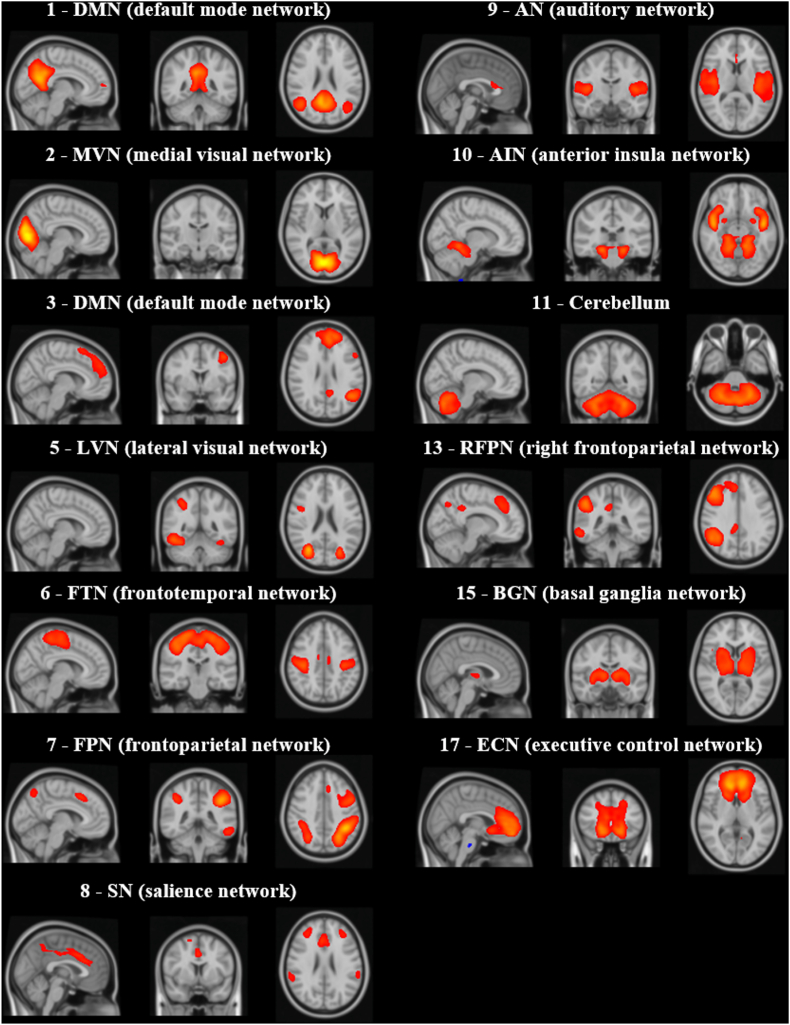
All identified components (IC) from the ICA analysis after datasets with motion larger than 1 voxel size have been removed. Artifacts have been removed from manual classification.

Totally 5 data sets were excluded from further analysis because of subject motion; 2 patients at time point 2, 2 healthy controls at timepoint 2 and 1 healthy control at time point 1. The ICA analysis resulted in 20 independent components (IC). The IC's were classified manually by considering the spatial maps, time courses and the power spectra of each component. In total, 7 components were discarded as artifacts such as motion, CSF, flow or WM-signal. The 13 remaining components were identified as the following resting state networks via visual inspection; the default mode network (DMN), the medial visual network (MVN), the lateral visual network (LVN), the frontotemporal network (FTN), the frontoparietal network (FPN), the salience network (SN), the auditory network (AN), the anterior insula network (AIN), the thalamus, cerebellum, right frontoparietal network (RFPN), the basal ganglia network (BGN) and the executive control network (ECN), all shown in Fig. 2.

**Fig. 3:**
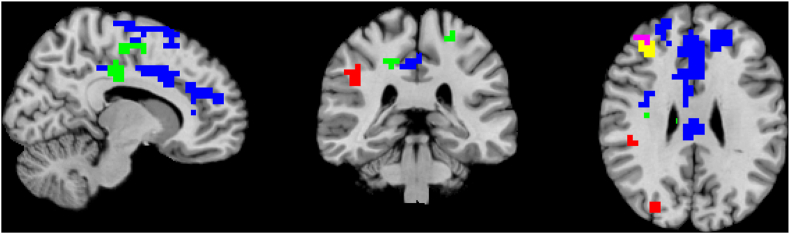
Clusters with significantly higher correlation to IC for patient group compared to control group. Correlation before PVT to the lateral visual network is shown in red, the salience network in blue, the auditory network in green, and the executive control network in yellow. After PVT correlation to the basal ganglia network is shown in cerise.

The IC spatial maps were then used in the dual regression to calculate the individual estimates for group- and time effects. Brain regions with a significant difference between patients and controls are reported in Table 2 and Fig. 3. After performance of the Psychomotor Vigilance Task (PVT), group differences in functional connectivity were detected only within the BGN. No significant time differences were detected. In addition, functional connectivity was not significantly correlated to self-assessment of depression (HADS) trait (MFI-20) and state (VAS-f) fatigue, nor trait (Pain during the last week) pain. Significant within group effects using self-assessed state pain (VAS-p) before and after MRI as regressors are reported in Table 3 below. There were no significant correlations between functional connectivity and changes in state pain and state fatigue from before to after MRI scanning.

**Table 2:** Clusters with significant different activity in the control group compared to the patient group before and after the Psychomotor Vigilance Task (PVT).

Brain region	IC	MNI coordinate (x, y, z)	Cluster volume (cm^3^)
**Significantly stronger correlation to IC for patient group compared to control group - before PVT**			
R SupraMarginal Gyrus	5 – Lateral Visual Network	46, −30, 32	1.152*
R Fusiform Gyrus		30, −74, 0	0.576*
R Precentral Gyrus		34, −10, 56	0.576*
R Superior Occipital Gyrus		26, −82, 28	0.256*
R Middle Occipital Gyrus		30, −82, 16	0.192*
L Superior Medial Gyrus	8 – Salience Network	−6, 54, 8	26.432*
R SMA		10, −2, 68	6.976*
L Superior Frontal Gyrus		−18, 42, 28	2.560*
L Inferior Parietal Lobule		−30, −42, 52	0.128
R SMA	9 – Auditory Network	14, −14, 56	3.584
R Inferior Frontal Gyrus		42, 38, −4	2.368*
R Superior Temporal Gyrus		42, −26, 0	0.704*
R Superior Orbital Gyrus		30, 58, −4	0.704*
L Putamen		−22, 22, 4	0.256
L Caudate Nucleus		−14, 10, 8	0.256
R Insula Lobe		34, −14, 28	0.256
L Insula Lobe		−26, 22, −4	0.192*
L Postcentral Gyrus		−22, −34, 60	0.192*
L Middle Cingulate Cortex		−14, −14, 48	0.128
L Precentral Gyrus	17 – Executive Control Network	−38, 2, 32	0.128
**Significantly stronger correlation to IC for patient group compared to control group - after PVT**			
R Precentral Gyrus	15 – Basal Ganglia Network	46, −10, 56	1.088
R Middle Frontal Gyrus		34, 34, 28	0.896*
R Middle Frontal Gyrus		30, 26, 20	0.128

Note: *Overlap between significant cluster and IC (within network).

**Table 3:** Clusters with significant group effect using the self-assessed state pain (VAS-p) before and after MRI as regressors.

Brain region	IC	MNI coordinate (x, y, z)	Cluster volume (cm^3^)
**Patients before PVT with self-assessed state pain before MRI as regressor**			
Right Precuneus	1 – Default Mode Network	14, −66, 28	12.480*
Left Precuneus		2, −66, 44	1.024*
Right Parahippocampal Gyrus		30, −30, −16	0.896*
Right Middle Cingulate Cortex		6, −34, 40	0.128*
Left Superior Frontal Gyrus	3 – Default Mode Network	−14, 62, 16	6.400*
Left SMA		−6, 22, 48	1.728*
Left Superior Medial Gyrus		−10, 50, 40	1.408*
Left Superior Medial Gyrus		−2, 34, 36	0.512*
Left Middle Orbital Gyrus		−2, 50, −4	0.448*
**Patients after PVT with self-assessed state pain after MRI as regressor**			
Left Postcentral Gyrus	1 – Default Mode Network	−46, −18, 32	0.128

Left Postcentral Gyrus	2 – Medial Visual Network	−42, −18, 52	4.608*
Left Middle Frontal Gyrus		−26, 50, 16	0.832
Left Superior Frontal Gyrus		−30, 58, 0	0.576
Left Thalamus	3 – Default Mode Network	−6, −18, 8	0.256

Right Precentral Gyrus	5 – Lateral Visual Network	34, −22, 60	1.600*
Right Superior Frontal Gyrus		26, −10, 56	0.896*
Right Superior Parietal Lobule		38, −46, 56	0.192*
Right Middle Cingulate Cortex	6 – Fronto-temporal Network	14, −22, 44	0.128*
Left Caudate Nucleus	7 – Fronto-parietal Network	−18, 18, 4	0.320*

Left Cuneus	8 – Salience Network	−18, −74, 36	1.920*
Right Cuneus		18, −66, 40	1.472*
Right Angular Gyrus		46, −46, 28	1.024*
Left Superior Temporal Gyrus		−58, −34, 20	0.512*
Left Superior Temporal Gyrus		−62, −42, 20	0.128*
Right Insula Lobe	10 – Anterior Insula Network	42, 18, −12	0.384*
Right Precuneus	13 – Right Fronto-parietal Network	6, −74, 36	0.512*
Right Middle Cingulate Cortex		10, −26, 36	0.256*
Left Middle Frontal Gyrus	17 – Executive Control Network	−26, 18, 48	0.512*
Left Anterior Cingulate Cortex		−2, 38, 24	0.256*
**Controls before PVT with self-assessed state pain before MRI as regressor**			
Left Postcentral Gyrus	8 – Salience Network	−38, −38, 64	0.128

Note: *Overlap between significant cluster and IC.

## 4 Discussion

In the present study we explored the neural correlates of pain and fatigue in patients with chronic pain using resting state functional magnetic resonance imaging. In addition to simply observing RST differences, the differences in resting state functional connectivity before and after a fatiguing PVT were investigated. Before the task, significant differences between patients with chronic pain and healthy controls were identified across several brain networks. These differences included connectivity between the DMN and the SN, regions within the DMN, the SN, and the executive control network, as well as the connectivity of sensory motor areas. Consistently, across all networks, functional connectivity was significantly increased in patients with chronic pain, as compared to healthy controls. These findings are consistent with previous research highlighting the role of networks such as the DMN, the SN, and sensory motor areas in chronic pain processing (Baliki et al., 2008; Hemington et al., 2016; Napadow et al., 2010). However, and possibly more interesting, after performance of a fatiguing vigilance task, most of these group differences were no longer statistically detectable. After the task, significant differences in connectivity between chronic pain patients and healthy controls were only present in connectivity between the basal ganglia network (BGN) and the right prefrontal cortex. These findings lead to two conclusions regarding the effects of including a fatiguing attention task to functional connectivity in patients with chronic pain: (1) Abnormal functional connectivity possibly associated with fatigue is observed only after the performance of a fatiguing task; and (2) abnormal functional connectivity related to pain are no longer statistically detectable after performance of a fatiguing attention task.

Regarding the first conclusion, the differences between groups in basal ganglia connectivity to the prefrontal cortex post task might be indications of altered functional resting state connectivity due to fatigue. Since there were no associations between basal ganglia connectivity and self-assessment of state or trait fatigue (discussed further below), nor any group x time state fatigue or functional connectivity interaction, this is however a tentative statement based on previous research in other clinical groups. The BGN, which has consistently been shown to be involved in motor control and reward processing, has in recent years been linked to fatigue and motivation (Chaudhuri and Behan, 2000, 2004), especially in patients with neurological disorders or injuries (Chaudhuri and Behan, 2004; Dobryakova et al., 2013; Schumann et al., 2025). The findings of the present study suggest that alterations in BGN connectivity may also contribute to the experience of fatigue in patients with chronic pain. Since effort-reward imbalances affecting motivation seems to be present in both fatigue (Dobryakova et al., 2013) and chronic pain (Porreca and Navratilova, 2017), it might be disrupted reward processing within the BGN that require an increased effort, in this case for a fatiguing vigilance task. The imbalance in effort and reward processing should thus be further investigated in future studies, both to investigate the experience of fatigue and to explore the potential for targeted interventions to modulate these networks (Borsook et al., 2016; Chaudhuri and Behan, 2000).

As for the second conclusion, the vanishing of significant differences post task within and between networks such as the DMN, the SN, the ECN, and sensory-motor areas, might indicate that the brain is not processing pain in the same manner as an effect of having been occupied with a task, or due to state fatigue. The DMN is typically active during rest and involved in self-referential thinking, and disruptions of connectivity might be a sign of lack of rest, such as ruminative thinking (Hogestol et al., 2019). The salience network (SN), including the anterior insula and anterior cingulate cortex (ACC), plays a crucial role in detecting, filtering and integrating salient stimuli, including pain signals (Seeley et al., 2007; Uddin, 2015). Both networks have been extensively studied in the context of chronic pain, and alterations in connectivity have been reported in various chronic pain conditions (Kim et al., 2017; Kucyi and Davis, 2015; Loggia et al., 2013; Napadow et al., 2010). A more normalized response post task, such as that in the present study, might be an indication that pain is not processed in the same way, possibly alleviating some of the pain experience. This is an interesting finding, since finding ways to alleviate the pain experience is central to these patients. Still, this is a tentative conclusion. Since the patient group actually showed increased experience of pain from before to after fMRI scanning, it might be that task-induced changes in functional connectivity might mask the effects of pain on functional connectivity. Future research on this matter is thus warranted.

In addition to these two main conclusions, the data from the present study also reveal some interesting findings regarding the relationships between self-assessments of fatigue and pain and functional connectivity during resting state. We investigated both state fatigability (as measured by changes in self-reported fatigue before and after the fatiguing PVT) and trait fatigue (as measured by the Multidimensional Fatigue Inventory-20, MFI-20) in relation to functional connectivity. Interestingly, while significant group differences in resting state functional connectivity were observed, there were no significant correlations to self-reported fatigue measures, neither before nor after the performance of the PVT. The lack of significant correlations between resting state functional connectivity and fatigue measures highlights the need for further research to elucidate the neural mechanisms underlying fatigue in patients with chronic pain. Results from previous studies have been quite mixed, where some have found significant correlations between resting state functional connectivity and clinical measures of fatigue or pain (Basu et al., 2018; Liu et al., 2020), while others have not (Hemington et al., 2016; Park et al., 2022). One possible explanation for this discrepancy could be the specific characteristics of the sample, which included only women with chronic pain, who were relatively young. This quite homogeneous sample may have led to less variability in fatigue levels, making it more challenging to detect significant correlations. Since age is an important mediator of neurocognitive dysfunction in patients with chronic pain (Oosterman and Veldhuijzen, 2016), the relatively young sample might also affect these results. Previous studies on mild traumatic brain injury have found changes in resting state functional connectivity more reliably related to fatigue (Bruijel et al., 2022; Nordin et al., 2016). This might indicate a more homogenous experience of fatigue in the TBI group than in patients with chronic pain. Indeed, the patients in this study all experienced chronic pain, but the pain had different etiologies. Future research could include more heterogeneous samples regarding age, but more homogenous samples regarding diagnosis, to better capture the neural correlates of fatigue in patients with chronic pain.

In contrast to self-assessment measures of fatigue, self-assessment measures of state pain did show significant associations to functional connectivity in the patient group. Not surprisingly, higher degree of state pain both before and after performance of the PVT was associated with stronger connectivity within the DMN and the SN. As noted above, both increased and decreased connectivity patterns within these networks have been observed depending on pain duration, diagnosis and intensity (Kucyi and Davis, 2015; Loggia et al., 2013; Napadow et al., 2010; Seeley et al., 2007; Uddin, 2015). In addition, the self-assessment of pain after performance of the PVT was also associated with increased connectivity within the medial visual network, the lateral visual network, the fronto-temporal and the fronto-parietal network. Several studies have found associations between pain and the fronto-temporal and fronto-parietal network in patients with chronic pain (Flodin et al., 2014; Jensen et al., 2012; Kim et al., 2017; Park et al., 2022), and the frontal functions do indeed seem central in the context of chronic pain (Ong et al., 2019). The associations between pain and the medial and lateral visual networks have been shown in some previous studies (Schrepf et al., 2018), but the cause of this finding should be further investigated in future studies.

The interaction between pain and fatigue is complex, with both symptoms often co-occurring in chronic pain conditions. The present study revealed that pain levels increased significantly in patients after the fatiguing task, whereas no such increase was observed in healthy controls. This finding is consistent with the hypothesis that chronic pain patients have a lower threshold for pain exacerbation, particularly when fatigued (Yamada et al., 2022). The significant interaction effect between time and group for pain levels suggests that fatigue may exacerbate pain perception in chronic pain patients, potentially through shared neural pathways (Apkarian et al., 2005; Van Damme et al., 2018). It is also possible that different brain networks or regions are involved in the modulation of pain and fatigue, or that the effects of fatigue on pain perception are mediated by other factors, such as emotional state or cognitive processing (Kuner and Flor, 2017; Ong et al., 2019).

This study has several limitations that need to be addressed when interpreting and generalizing the results. While the sample of patients were ecologically relevant, since all patients were seeking specialized pain rehabilitation, the sample consisted of only women. Although a majority of patients living with chronic pain are female, this means that the results are not directly generalizable to all patients. In addition, the sample was quite young (mean age 33 years) in comparison with the general chronic pain population, further limiting generalizability. Furthermore, the healthy control group had significantly higher number of years of education, which could explain some differences between groups. However, adjusting for education in the RST analyses did not alter the results to any notable degree. As in most functional brain imaging studies, the total sample size was quite small, affecting power, which might have rendered some type II-errors. Critical for this study, this might have affected the possibility to discover significant associations between BGN functional connectivity and self-assessment of fatigue. The low sample size has also affected the possibility of adjusting for relevant covariates, such as depression, age, and education in several analyses. The resting-state acquisition time was relatively short, which limited the feasibility of volume censoring approaches without substantially reducing the remaining data available for connectivity analyses. Therefore, a dataset-level exclusion strategy based on visual inspection of translational and rotational motion parameters was applied.

## 5 Conclusions

Our findings suggest that after the performance of a fatiguing attention task, only abnormal functional connectivity related to fatigue is detectable in patients with chronic pain. In addition, alterations in key brain networks connected to pain, such as the default mode network and the salience network, were no longer statistically detectable after performing a fatiguing attention task in patients with chronic pain, indicating an altered pain experience. However, the lack of significant correlations between resting state functional connectivity and fatigue measures underscores the complexity of these symptoms and the need for further research. Thus, these findings should be considered preliminary, and the interaction between pain and fatigue warrants further investigation, particularly in terms of identifying shared neural pathways between motivation/reward, pain and fatigue.

## Funding

This study was supported by 10.13039/501100004047Karolinska Institutet, Department of Clinical Sciences, the 10.13039/100009389Promobilia Foundation (no. A22056), by the research and development fund granted by Västerbotten County Council, and through a regional agreement between Umeå University and Västerbotten County Council (ALF).

## CRediT authorship contribution statement

**Nils Berginström:** Conceptualization, Data curation, Formal analysis, Funding acquisition, Investigation, Methodology, Project administration, Visualization, Writing – original draft, Writing – review & editing. **Marika C. Möller:** Conceptualization, Data curation, Formal analysis, Funding acquisition, Supervision, Writing – review & editing. **Anna Holmqvist:** Conceptualization, Funding acquisition, Investigation, Project administration, Writing – review & editing. **Monika Löfgren:** Conceptualization, Writing – review & editing. **Britt-Marie Stålnacke:** Conceptualization, Funding acquisition, Supervision, Writing – review & editing. **Love Engström Nordin:** Conceptualization, Data curation, Formal analysis, Methodology, Writing – original draft, Writing – review & editing.

## Declaration of competing interest

The authors declare no competing interests.

## Data Availability

Data will be made available on request.
